# Identification and virtual screening of novel anti-inflammatory peptides from broccoli fermented by *Lactobacillus strains*

**DOI:** 10.3389/fnut.2022.1118900

**Published:** 2023-01-11

**Authors:** Yao Li, Xinchang Gao, Daodong Pan, Zhu Liu, Chaogeng Xiao, Yongzhao Xiong, Lihui Du, Zhendong Cai, Wenjing Lu, Yali Dang, Xiuzhi Zhu

**Affiliations:** ^1^State Key Laboratory for Managing Biotic and Chemical Threats to the Quality and Safety of AgroProducts, College of Food and Pharmaceutical Sciences, Ningbo University, Ningbo, Zhejiang, China; ^2^Department of Chemistry, Tsinghua University, Beijing, China; ^3^Zhejiang Institute for Food and Drug Control, Hangzhou, Zhejiang, China; ^4^Zhejiang Academy of Agricultural Sciences, Hangzhou, Zhejiang, China; ^5^Department of Gynecology and Obstetrics, The Second Affiliated Hospital of Zhejiang Chinese Medical University, Hangzhou, Zhejiang, China

**Keywords:** fermented broccoli, anti-inflammatory peptides, peptidomics, virtual screening, *Lactobacillus*

## Abstract

*Lactobacillus strains* fermentation of broccoli as a good source of bioactive peptides has not been fully elucidated. In this work, the peptide composition of broccoli fermented by *L. plantarum* A3 and *L. rhamnosus* ATCC7469 was analyzed by peptidomics to study the protein digestion patterns after fermentation by different strains. Results showed that water-soluble proteins such as rubisco were abundant sources of peptides, which triggered the sustained release of peptides as the main target of hydrolysis. In addition, 17 novel anti-inflammatory peptides were identified by virtual screening. Among them, SIWYGPDRP had the strongest ability to inhibit the release of NO from inflammatory cells at a concentration of 25 μM with an inhibition rate of 52.32 ± 1.48%. RFR and KASFAFAGL had the strongest inhibitory effects on the secretion of TNF-α and IL-6, respectively. At a concentration of 25 μM, the corresponding inhibition rates were 74.61 ± 1.68% and 29.84 ± 0.63%, respectively. Molecular docking results showed that 17 peptides formed hydrogen bonds and hydrophobic interactions with inducible nitric oxide synthase (iNOS). This study is conducive to the high-value utilization of broccoli and reduction of the antibiotic use.

## 1. Introduction

Inflammation is the aggregate immunoreaction of the body to certain irritants, such as microbiological pollution, and oxidative stress (1). However, long-term inflammation may provocate diabetic mellitus, fat, cardiovascular disease, inflammatory bowel disease, neural inflammation, and other ailments (2). Adjusting mass propagation, swallowing of macrophages, and secretion of pro-inflammatory cytokines can effectively treat inflammation (3). In general, endocellular nitric oxide (NO) keeps stable at low levels for a long time. However, under the stimulation of various inflammatory conditions, iNOS catalyzes L-arginine oxidation to produce a large amount of NO, which results in tissue damage (4). Inhibition of iNOS could effectively control the inflammatory response in various pathological conditions (5). Therefore, inhibition of iNOS activity has become an important method of treatment of inflammatory diseases. Nevertheless, taking antiphlogistic drug medicines for a long time can easily lead to gastrointestinal diseases, renal insufficiency, and other side effects (6). Moreover, the misuse of antibiotics to treat inflammation caused by bacterial infections can easily make the bacteria resistant, making the infection more difficult to treat and ultimately likely leading to previously preventable deaths (7). In consequence, the evolution of innoxious and easily absorbed natural anti-inflammatory agents has become a research focus.

Fermentation, a microbial physiological process is attracting extensive concern for its effect on improving functional properties (8). Lactic acid bacteria (LAB) are the main microorganisms in fermented food, which have important effects on the quality and physiological activity of fermented food. The main genera of LAB such as *Enterococcus, Lactobacillus, Streptococcus*, and *Tetragenococcus* have been isolated from fermented foods worldwide (9). Besides the energy and nutritional properties of fermented filtrate, LAB fermentation as an excellent source of bioactive peptides is also well known (10). Many peptides with anti-inflammatory activity or other biological activity have been obtained from various fermented foods, such as fermented milk (11), fermented soybean (12), oyster (13), etc. Fermentation of unprocessed protein-rich organisms has become an important strategy for the production of bioactive peptides in the biotechnology industry.

Broccoli is a variety of *Brassica* species in Cruciferae with green flower balls as products, which is a favorable source of health-promoting nutrients. The most usual bioactive compounds in broccoli are considered to be secondary metabolites such as sulforaphane, phenolic acid, and flavone (14). Therefore, previous research always concentrated on the changes in these metabolites during the fermentation process (15, 16). But few studies have explored the peptide changes of broccoli during fermentation. Plant proteins are modified differently during fermentation to generate a series of hydrolysates with variant bioactivities, such as peptides. It is noteworthy that these hydrolysates are significant for the functions of fermented foods. Among them, bioactive peptides exhibit antihypertensive, hypoglycemic, cholesterol-lowering, anti-inflammatory, and other physiological activities (17). Enzymatic hydrolysis or fermentation to obtain active peptides has been widely used in food processing (18). Broccoli protein could be effectively degraded by its endogenous protease and exogenous protease secreted by microorganisms (19). It is a pity that there are few studies on the identification of peptides and active peptides of LAB fermented broccoli. Previously, active peptides with hypoglycemic and antihypertensive properties have been identified from broccoli, but peptides with anti-inflammatory properties have not been identified (20). Meanwhile, the traditional separation and purification of bioactive peptides are very cumbersome and time-consuming (21). The combination of peptidomics and virtual screening techniques (bioinformatics, molecular docking, etc.) can be used as a tool for the rapid screening of bioactive peptides, thereby effectively improving experimental efficiency.

In this paper, high-flux peptidomic methods were used to study the peptide profiles of fermented and unfermented broccoli of *L. plantarum* A3 and *L. rhamnosus* ATCC7469 and to quickly screen anti-inflammatory peptides. Finally, the molecular mechanism of anti-inflammatory peptides to the iNOS receptor was evaluated. This research will contribute to the high-value utilization of broccoli and provide a new understanding of increasing the functional value of broccoli fermented by LAB.

## 2. Materials and methods

### 2.1. Materials

The stems and leaves of broccoli (*Brassica oleracea var. italica*) were obtained from Wumei Market (Ningbo, China). *Lactobacillus plantarum* A3 and *Lactobacillus rhamnosus* ATCC7469 were preserved in our laboratory and used in this study. Peptides from broccoli were synthesized by GL Biochemical, Ltd. (Shanghai, China).

RAW264.7 cells were obtained from the Procell (Wu Han, China). Dulbecco's modified Eagle medium (DMEM), Penicillin/streptomycin solution (P/S), and fetal bovine serum (FBS) were obtained from HyClone (Logan, UT, USA). Lipopolysaccharides (LPS), CCK-8 kits, and nitric oxide test kits were provided by Bey time (Shanghai, China). TNF-α and IL-6 ELISA kits were purchased from Multi Sciences (Hangzhou, China). Pierce^TM^ Quantitative Peptide Assays Kit was purchased from Thermo Fisher Scientific (NY, USA). Other chemicals and reagents are analytically pure.

### 2.2. Sample preparation

The fresh stems and leaves of broccoli were washed with ultrapure water and juiced, and solid particles were separated by gauze filtration. The juice was transferred into a 200 ml conical flask and autoclaved at 121°C for 15 min to eliminate the influence of endogenous bacteria.

### 2.3. Broccoli fermentation

The two strains of lactic acid bacteria were activated in a constant temperature and humidity incubator at 37°C. The activated strains were centrifuged for 5 min at 10,000 × g. And then the precipitate was dissolved in 100 mL of broccoli juice. The initial cell density of the final solution was 10^7^ CFU/mL and cultured at 37°C for 24 h. After fermentation, the supernatant was centrifuged and stored at −80°C. All fermentation experiments were repeated three times.

### 2.4. Peptide extraction

The peptides were rapidly extracted from the hydrolysate of broccoli by ultrafiltration centrifuge tube (*Mw* = *10 kDa*, Merck KGaA, Darmstadt, Germany), freeze-dried, and stored at −80°C for further analysis. According to the manufacturer's instructions, the extracted peptides were further cleaned and concentrated using Oasis HLB cartridges (Waters, Milford, MA, USA), which were modified by our research team to remove salt from the sample. The specific program is shown in Supplementary Table S1. The peptide detection kit is used to determine the peptide content in the sample.

### 2.5. Peptide identification by LC-MS/MS

The detection system was combined with the Thermoelectric Easy-nLC 1200 (Thermo Scientific, P/N LC140) and the orbital trap Exploris 480 (Thermo Scientific, P/N BRE725533) for peptide identification. All mass spectrometer parameters were followed by Zhang et al. (22). The peptide was captured by a capture column (PepMap C18, 100 μm × 25 cm) for 3 min, and then the peptide was separated by gradient elution chromatography on a nano-upgrade analytical column (PepMap C18, 75 μM × 25 cm). Mobile phase A: 0.1% formic acid aqueous solution, mobile phase B: 0.1% formic acid acetonitrile solution; the separation gradient of mobile phase B increased from 5 to 30% within 60 min. The chromatographic flow rate was 300 nL/min. The sample volume was 5 μL; the column temperature was 55°C.

The full scan MS scan range was set to m/z 100–1,600, resolution 70,000 (m/z 200). The maximum ion introduction time was set to 50 ms, the Auto Gain Control was set to 5 × 10^5^, the intensity of the first 15 parent ions in the high energy collision dissociation experiment, and the scanning rate was executed at 17,500. Based on the parent ion mass charge ratio automatic control scanning range, the minimum scanning range was set to m/z = 100, up to 2,000. When MS/MS analysis was performed, the minimum ionic strength was set to 13,000, the maximum ion introduction time was set to 100 ms, and the Auto Gain Control was controlled to 2.0 × 10^5^. The parent ion error tolerance was set at 1.6 Da. Finally, ions with charges of 1, 2, 3, and 4 were collected, and the exclusion was set to MS/MS analysis of the parent ion within 10 s, and then 40 s, 30% collision energy was excluded.

PEAKS Studio 8.5 (Bioinformatics Solutions, Waterloo, Canada) was used for mass spectrometry data analysis and broccoli protein database matching (https://www.ncbi.nlm.nih.gov/data-hub/taxonomy/tree/?taxon=36774). The identified protein sequence data were retrieved from the UniProt KB database, and the retrieved sequences were searched locally for homology sequences and their functional annotations. PEAKS Studio was used to quantify the abundance of identified peptides. The false positive rate (FDR) of peptide identification was set at 1%. For the results of *de novo* sequencing, ALC (%) is the full mean local confidence. *De novo* sequencing data are more reliable when the ALC was greater than 80%, and these data were retained. The BIOPEP database was used to retrieve bioactive peptides and identify peptides from samples. Peptide mapping was done using the online tool Peptigram (23). The potential activity of each peptide was identified using the PeptideRanker tool.

### 2.6. Identification and function prediction of broccoli peptides

PeptideRanker scores above 0.5 were designated as potential bioactive peptides, where the higher the score, the higher probability of the peptide being bioactive. The anti-inflammatory peptides were predicted by AIPpred (24) and Antiinflam (25). The anti-hypertensive peptides were forecast by AHTpin (26), and mATPred (27). The pro-inflammatory activity of the peptides was predicted by VaxinPAD (28). AntiCP 2.0 (29) had access to predict anticancer peptides. The toxicity and major properties of peptides were identified using the ToxinPred13 and Innovagen platforms (http://www.innovagen.com/proteomics-tools).

### 2.7. Determination of the anti-inflammatory activity of broccoli peptides

#### 2.7.1. Albumin denaturation assay (AI)

The anti-inflammatory properties of broccoli hydrolyzates were preliminarily determined by the albumin denaturation method (30). The reaction mixture was composed of 5 ml bovine serum albumin (1%, pH = 6.3), 0.5 mL broccoli sample solution, incubated at 37°C for 20 min, and then incubated at 51°C for 20 min. The absorbance at 660 nm was recorded using Tecan microplate reader (Infinite M2000 Pro, Switzerland). Diclofenac sodium was used as the positive control.

#### 2.7.2. Determination of inflammatory factors

RAW264.7 cells were cultured in DMEM medium containing 10% FBS and 1% double antibody at 37°C and 5% CO_2_ incubator. CCK-8 reagent was used for cell viability assay.

RAW264.7 cells (3 × 10^6^ cells / mL) seeded into 12-well plates were pretreated with various concentrations of broccoli hydrolyzates or synthetic peptides for 2 h before stimulation by LPS (200 ng/mL). NO was determined using Griess reagent. TNF-α and IL-6 were measured by ELISA kits according to the manufacturer's instructions. More details of the assay are shown in Supplementary Table S2.

### 2.8. Synthesis of specific sequence peptides

Peptide sequences from broccoli were synthesized by a solid-phase peptide program using a 9-fluorenyl methoxycarbonyl (Fmoc) protected amino acid synthesis method and an AAPPTEC 396 Automatic Peptide Synthesizer (Advanced Automated Peptide Protein technologies, USA). The purity of synthetic peptide was determined by HPLC to be more than 98%.

### 2.9. Molecular docking with iNOS

Discovery Studio software (Neo Trident Technology Ltd., Shanghai, China) was used for molecular docking. The peptide structure was drawn by Chem Draw Prime 19.1 software. The crystal structure of iNOS (PDB ID 3E6T) complexed with AR-C118901 was prepared. Molecular docking was referenced by Wang et al. (31) with some modifications. Before docking, the protein crystals were treated as required, water and cofactors were removed, hydrogen was added, and other programs were completed as required. Create a receptor region with a radius of 20Å. The docking energy score and interaction force type evaluate the results.

### 2.10. Statistical analysis

All experiments were repeated 3 times, and the results were expressed as mean ± standard deviation. Excel, SPSS Statistics 23.0 (IBM, NY, USA), and GraphPad Prism 9.0 (GraphPad Software, CA, USA) were used to process the data. *P* < 0.05 was considered significant.

## 3. Results and discussions

### 3.1. Proteomics analysis of peptides

#### 3.1.1. General analysis

As a branch of proteomics, peptidomic analysis has access to use to identify the peptide of samples (32). After the raw data processing, 17,581 peptides were retained from all broccoli samples. Among them, 5,901 peptides were obtained by *de novo* sequencing. About 5,081, 6,359, and 4,894 peptides belong to 2,300, 1,982, and 2,126 proteins for broccoli from NC (broccoli hydrolysate fermented for 0 h), *L. plantarum* A3, and *L. rhamnosus* ATCC7469, respectively. Compared with before fermentation, the number of broccoli proteins decreased after fermentation, which indicated that a small cluster of proteins was degraded by *Lactobacillus*. Totally, 595 peptides were commonly found in each broccoli sample, and they belong to 559 accession proteins (Supplementary Figure S1). The major broccoli proteins were identified, such as jacalin (jacalin-related lectin 34-like isoform X1 and X2), photosystem I, rubisco, ribosomal protein, and other proteins. These identified proteins are different from previous studies (20).

Although most proteins were identified in all samples, there were still differences: 1,754 proteins were absent in NC, while 2,072 and 2,332 proteins were detected in *L. plantarum* A3 and *L. rhamnosus* ATCC7469, respectively. The difference may be due to the different processing methods of samples, and the extracellular enzyme of *Lactobacillus* is more complex than a single enzyme. However, their differences at the peptide level are considerable. A total of 6,368 peptides were identified in NC, 9,570 in *L. plantarum* A3, and 8,203 in *L. rhamnosus* ATCC7469.

**Figure 1:**
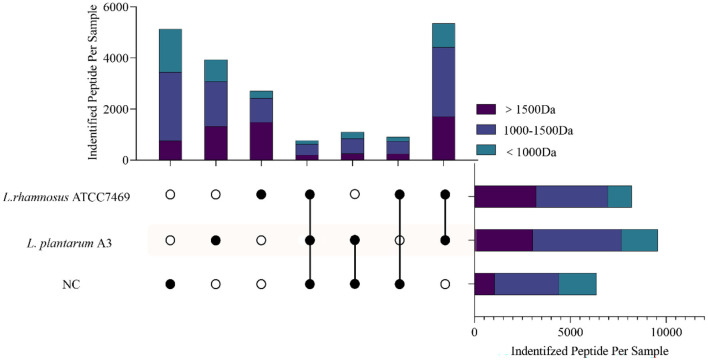
The cross plot of peptides identified in broccoli stems and leaves fermentation samples (NC, fermentation for 0 h; *L. plantarum A3, Lactobacillus plantarum* fermentation; *L. rhamnosus* ATCC7469, *Lactobacillus rhamnosus* fermentation). Different colors on the strip represent different molecular weights belonging to each cross-group peptide.

Figure 1 shows the intersection of the peptides identified from the three samples. There were some differences in the peptide content of broccoli hydrolysate before and after fermentation. The most abundant peptide was from the hydrolysate fermented by *L. plantarum* A3 (16.23%), followed by *L. rhamnosus* ATCC7469 (11.21%). Interestingly, 5,130 peptides (accounting for 21.22% of the total) only existed in unfermented samples and had higher exclusive peptides compared with other groups. During sample treatment, high temperature and pressure might promote the release of peptides (33).

The restricted proteolytic ability of microorganisms is related to the unique sequence and functional peptide fragments produced by the proteolysis of specific residues (34). This indicates that even though different microorganisms of the same genus have different hydrolysis mechanisms, they can still hydrolyze target proteins at the same site and produce peptides with the same sequence (35). It is noteworthy that, all the peptides identified in the three samples were analyzed. As hydrolysates of proteins, amino acids and peptides undergo secondary hydrolysis during fermentation and are consumed by microorganisms (36). Compared with the NC, not only the total number of peptides increased after fermentation, but also the identified peptides were mainly distributed in small molecular weight (< 1,000 Da), which was significantly higher than NC. This may indicate that *Lactobacillus* has a good ability to hydrolyze broccoli protein, and can hydrolyze the peptides existing in the NC, resulting in more small molecule active peptides. This proved that *Lactobacillus* fermentation could be a good method for the hydrolysis of plant protein to produce peptides.

#### 3.1.2. Changing of peptides during fermentation in broccoli

**Figure 2:**
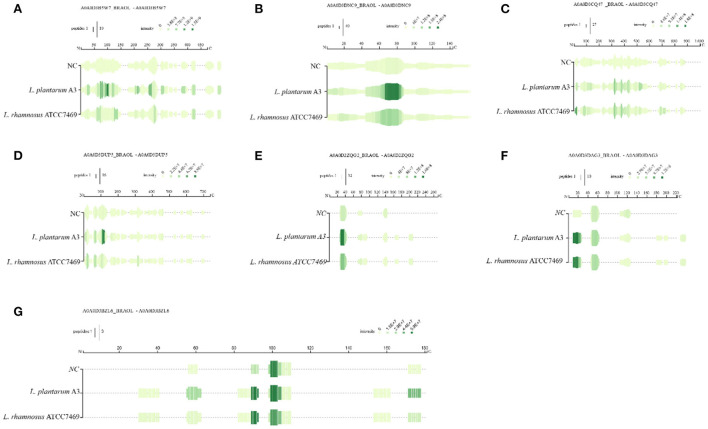
Mapping of the identified peptides for the three samples on the sequences of rubisco **(A)** photosystem-I protein **(B)** transketolase **(C)** jacalin **(D)**, rubisco **(E–G)**.

During the fermentation process, microorganisms secrete a succession of enzymes that convert macromolecular organic matter in the substrate into small molecules (37). *Lactobacillus plantarum* and *Lactobacillus rhamnosus* are typical LAB for fermentation. It is characterized by high proteolytic activity, extracellular cell wall serine protease, and intracellular peptidase has an extensive range of specificness, including aminopeptidase, carboxypeptidase, and dipeptidase (38). During the fermentation of broccoli, some water-soluble proteins were degraded by LAB, and these proteins were found to be regulatory enzymes in plant physiological metabolism. The peptides on the primary sequence of the identified protein were located and four of the most changed proteins were projected, shown in Figure 2. Among them, ribulose bisphosphate carboxylase was abundant in all samples and increased significantly after fermentation with normalized intensity of 10^8^-10^9^ (Figure 2A).

This indicates that ribulose bisphosphate carboxylase can be hydrolyzed by microorganisms to become a nitrogen source for its growth and reproduction during microbial proliferation. This result explains the mobile equilibrium of components in the broccoli fermentation broth. Photosystem-I protein same goes for 10^8^ (Figure 2B). After fermentation, the sequence almost completely covered the entire protein, and the intensity increased significantly, and the intensity change was more significant in the 60–90 region. In addition, the strength of overlapping peptides at a certain position is proportional to the green depth. The peptides covered in the NC group were the least and the intensity was low, indicating that fermentation could promote protein release. Similar results apply to peptides derived from jacalin and transketolase. The peptides derived from these proteins have a low abundance in NC samples, which may be because these proteins have good thermal stability and are not easy to thermal decomposition. After fermentation, the primary sequence continuously covers the entire mature protein. Only the abundance of peptides at specific locations has been greatly improved. Rubisco is the most abundant protein in plants. Figures 2E–G shows the difference in peptide formation after the fermentation of three different types of rubisco proteins. Discontinuous coverage in the NC sample is then increased after fermentation and extended to the C-term region. However, there are large gaps between sequences in these regions. Even if they have similar cleavage sites at some positions, the relative strength of the peptides they produce is significantly different. Between 20–40 sites (Figures 2E, F), after fermentation, the green becomes deeper and the strength is significantly improved. In addition, the coverage of *L. plantarum* A3 was wider than that of *L. rhamnosus* ATCC7469 to produce peptides with high intensity. For photosystem-I protein, it was almost completely covered, and its strength was significantly improved after fermentation. After fermentation, its abundance is greatly increased, indicating that microorganisms can also use these two proteins for life activities. Other researchers reported similar results, and they found the most abundant category number was the 'catalytic activity' category of camellia proteins (36). This may provide evidence that the functional properties of proteins in broccoli are highly correlated with substrates.

To better understand the correlation between the digestion process of broccoli protein before and after fermentation and the appearance of its derived peptides, the peptide concentration was normalized and clustered. To reduce the effect of missing values, peptides present in all samples are clustered first. In addition, the second cluster analysis was performed on all peptides that existed in the fermentation group but did not exist in the NC group. Cluster analysis of the co-existing peptides between samples showed that the content of co-existing peptides before and after fermentation was different (Supplementary Figure S2). The small branch distance between *L. plantarum* A3 and *L. rhamnosus* ATCC7469 indicated that the similarity of peptide content between them was high. Additionally, quantitative results showed that the content of most peptides increased significantly after fermentation compared with NC group. In addition, the quantitative levels of broccoli peptides after fermentation with two different strains (FC ≥ 2 or ≤ 0.5, *p* < 0.5, *n* = 260) were compared, which were not present in NC (Supplementary Figure S3). Compared with *L. plantarum* A3, a total of 75 peptides were significantly enhanced and 185 peptides were significantly reduced. Compared with *L. plantarum* A3, a total of 75 peptides were significantly enhanced and 185 peptides were significantly reduced. This shows that there are significant differences in peptide release ability between different strains. Similarly, peptides from ribiso are the main peptides, but there are also peptides from ATP synthase subunit beta, Jacalin, and other small proteins. The hydrolysis of these proteins may contribute to the release of active peptides (36).

#### 3.1.3. Bioinformatics analysis for the potential bioactivity of broccoli peptides

**Figure 3:**
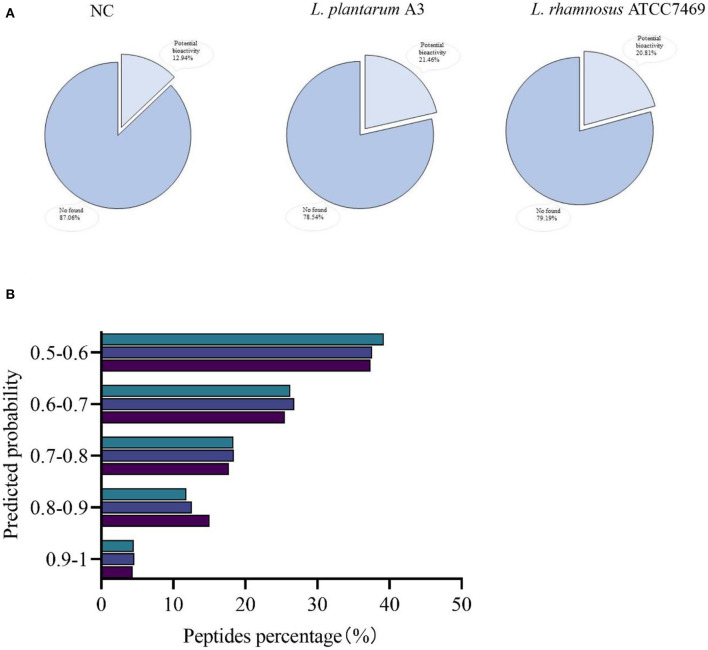
Ranked according to the predicted probability of potential biological activity of peptides. Coverage **(A)** and classified **(B)**.

All peptides were searched in the BIOPEP-UWM database to appraise potential bioactive peptides. Unfortunately, no peptides completely matched with the database were found in this study. Most of the bioactive peptides in BIOPEP database were 2 to 3 amino acids. In our study, most peptides were found to be more than 4 peptides. Therefore, we used PeptideRanker online tool to predict all peptide activity detected in our study (Figure 3). In PeptideRanker, any peptide predicted to exceed 0.5 threshold is considered to have biological activity. Totally, 824 (12.94%), 2,060 (21.46%), and 1,707 (20.81%) peptides with a score of more than 0.5 were found in broccoli from NC, *L. plantarum* A3, and *L. rhamnosus* ATCC7469, respectively (Figure 3A).

The distribution of these potentially bioactive peptides is shown in Figure 3B. The percentage of peptides scored between 0.9 and 1.00 in broccoli from *L. plantarum* A3, and *L. rhamnosus* ATCC7469, was 4.37, 4.61, and 4.51%. In addition, the relative abundances of the potentially active peptides in the three samples were calculated. The standardized intensity of the samples from NC was found 7.98 ×10^8^, the relative abundance of *L. plantarum* A3 was 6.92 × 10^10^, and *L. rhamnosus* ATCC7469 was 2.56 × 10^10^. Compared with unfermented broccoli, both the proportion and relative abundance of potential bioactive peptides increased significantly after LAB fermentation. And there are differences in strains, *L. plantarum* A3 fermentation than *L. rhamnosus* ATCC7469 fermentation is more obvious. Wei et al. (39) had similar research results to ours. The diversity and abundance of bioactive peptides increased significantly after fermentation of their furu samples, probably because most of the enzymes secreted during fermentation degraded the proteins to produce more bioactive peptides. This result showed that microbial fermentation could produce bioactive peptides by hydrolysis of proteins from broccoli, thereby improving their potential biological activities.

### 3.2. Functional prediction of bioactive peptides

Bioinformatics, which relies mainly on mathematical and statistical models to accurately predict the likely biological activities of individual peptides, seems to be useful for screening large numbers of peptides and assessing certain functions (35). The broccoli was fermented by LAB, and the protein was degraded by an extracellular protease to produce bioactive peptides, which gave it higher biological activity. To better study the bioactive peptides from broccoli, we selected peptides with PeptideRanker score > 0.5 for functional analysis. Anti-inflammatory, anti-hypertensive, anti-cancer, DPP IV inhibitory, and immunoregulatory activities were screened. The results showed that 84.16% of all predicted peptides had anti-inflammatory effects. In addition, 63.95% of the peptides were predicted to be antihypertensive peptides, 64.56% were considered to have anticancer activity, 47.70% were considered to be potential DPP IV inhibitors, and 7.43% of the peptides were predicted to have immunoregulatory activity (see Supplementary material B).

Computer screening of anti-inflammatory properties of peptides using network servers shows that there are a large number of anti-inflammatory peptides with different prediction probabilities in each hydrolysis station service. About 37.7% of the peptides in the hydrolysates fermented by *L. plantarum* A3 were predicted to be anti-inflammatory. In the hydrolysates fermented by *L. rhamnosus* ATCC7469, 31.83% of the peptides were anti-inflammatory, while only 14.59% of the NC were thought to be anti-inflammatory peptides. These predicted anti-inflammatory peptides contain one or more hydrophobic amino acids at the C- or N-terminus.

Besides, most peptides contain positively charged amino acids. Research has shown that the overall positive charge of peptides may be a chemokine, but their location is not fixed (40). Peptides with highly alkaline N-terminal or N-terminal Arg residues can bind to LPS released by bacteria, thereby blocking the inflammatory response.

To reduce the workload, of all the predicted anti-inflammatory peptides, 395 peptides (PeptideRanker score > 0.8) were retained for molecular docking after the removal of repetitive peptides and peptides longer than 15 amino acids (see Supplementary material B). These potential anti-inflammatory peptides are derived from F-box domain proteins, protein kinase domain-containing proteins, ribosomal proteins, rubisco, and other proteins, including uncharacterized proteins. Among them, the protein sources of most anti-inflammatory peptides have not been characterized. Of the identified proteins, proteins containing the F-box domain are the most abundant source of anti-inflammatory peptides among the three hydrolysates, followed by proteins containing the protein kinase domain. Some peptides were also identified from ribosomal protein and rubisco. F-box protein plays an important role in plant growth and development. Rubisco is the most abundant enzyme in plants and the most extensive source of plant bioactive peptides (41). Ribosomal proteins are also considered an important source of active peptides (42). Water-soluble proteins such as F-box domain protein, ribulose diphosphate carboxylase large chain, and 60S ribosomal protein L13a have a positive effect on the release of anti-inflammatory peptides. This indicates that potential anti-inflammatory peptides can be obtained from broccoli proteins, and increasing the content of active peptides in fermented broccoli will facilitate the degradation of these proteins, thereby improving the bioavailability of broccoli.

### 3.3. Determination of broccoli peptides activity

Inflammation is an important response of the body to foreign microbial infection and repair damage. But overage and unbounded inflammatory variations often lead to chronic maladies (1). Tissue protein degeneration is one of the causes of inflammation and arthritis (30). Therefore, ground on the prediction results of bioactive peptides, the albumin denaturation test (AI) was used to determine the anti-inflammatory activity of broccoli hydrolysate. The results of AI denaturation were expressed as diclofenac sodium equivalent (DSE) μg/mL, as shown in Table 1.

**Table 1:** Broccoli hydrolysate inhibition albumin denaturation test.

**Samples**	**AI(μg/mL of DSE)**
NC	280.89 ± 3.1646^c^
*L. plantarum* A3	1,798.39 ± 1.0014^a^
*L. rhamnousus* ATCC7469	1,403.744 ± 5.8357^b^

Lowercase letters a, b, and c indicate significant differences between samples (*p* < 0.05).

**Figure 4:**
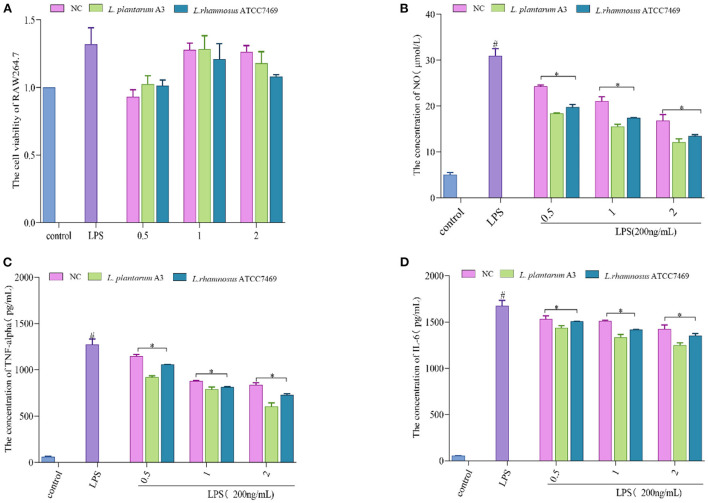
**(A)** Effect of different concentration samples (mg/mL) on the viability of RAW264.7 cells. **(B–D)** represent the effects of different concentrations of samples on the secretion of NO, TNF-α, and IL-6 in RAW264.7 cells after LPS stimulation. #, LPS is significantly different from control; *, The difference between the experimental group and the LPS group was significant (*P* < 0.05).

The results showed that different broccoli hydrolysates could inhibit albumin denaturation. But there are significant differences between them. Compared with NC, the anti-inflammatory potential of the other two hydrolysates was significantly enhanced. Among them, the hydrolysate of *L. plantarum* A3 had the highest anti-inflammatory activity, followed by *L. rhamnosus* ATCC7469, with DSE values of 1,798.39 ± 1.14 and 1,403.744 ± 5.84 μg/mL, respectively. In addition, the anti-inflammatory ability of the samples was further determined by LPS-induced RAW264.7 inflammatory cell model. It found that all three hydrolysates could promote the proliferation of macrophages, and increased first and then decreased with the increase of concentration. However, no significant toxic effects were observed at high concentrations (Figure 4A).

Treatment of macrophages with LPS causes over-immunity, producing large amounts of inflammatory factors (43, 44). Mouse macrophage RAW264.7 cells are commonly used to assess anti-inflammatory properties because their high sensitivity to external stimuli leads to the secretion of inflammatory cytokines (45). The data showed that all of them could prominently inhibit the production of NO, TNF-α, and IL-6. Among them, *L. plantarum* A3 had the strongest anti-inflammatory effect. At the maximum concentration (2 mg/mL), the inhibition rates of NO, TNF-α, and IL-6 were 60.52 ± 2.32%, 55.09 ± 2.87%, and 28.29 ± 1.57%, respectively (Figures 4B–D). This further indicates that LAB fermentation could improve the functional activity of broccoli. This may be because fermentation promotes the release of bioactive peptides and other active substances in broccoli to enhance anti-inflammatory activity. The results also strongly suggested that starter selection is still a major and important step in the development of fermentation-based biological processes. The research of Sun et al. (46) also proved this point. They found that different lactic acid bacteria increase the antioxidant and hypoglycemic activity of pumpkin juice by releasing bioactive substances such as phenols. Among them, *L. plantarum, L. acidophilus*, and *L. helveticus* had the strongest antioxidant capacity after fermentation.

### 3.4. Molecular docking with iNOS

A total of 72 known active anti-inflammatory peptides were docked with iNOS receptors as the positive control (Supplementary Table S3). The docking energy results showed that the docking energy of short peptides was generally higher than that of long peptides. Since the long-chain peptide has side-chain radicals, it increases the possibility of combining with the reactive site of the receptor. Furthermore, long-chain peptides may have greater flexibility and shorter docking distances to receptors (47). Hence, when utilizing molecular docking to determine whether the peptide has anti-inflammatory activity, peptide chain length and docking energy should be combined for evaluation. In this study, 395 potential anti-inflammatory peptides screened from broccoli were docked by DS software.

Molecular docking results based on reported anti-inflammatory peptides, a total of 251 peptides were found to bind to iNOS. Among them, the samples fermented by *L. plantarum* A3 had the most peptides that could bind to iNOS, followed by the samples fermented by *L. rhamnosus* ATCC7469. As a result, 13 unique peptides in NC products were also found. Besides, 85 and 56 unique peptides were found in the hydrolysates of *L. plantarum* A3 and *L. rhamnosus* ATCC7469, respectively. These peptides are predicted to be non-toxic, providing the possibility for safe use. Wen et al. (48) predicted four anti-inflammatory peptides using the computer prediction method of Net MHC II pan 4.0. However, almost all anti-inflammatory peptides screened by this method are peptides longer than 10, which may be because MCII has an open binding cleft and interacts mainly with long peptides of 13–25 lengths, which may be a defect of the model (49). The use of machine learning and docking of iNOS receptors in this work avoids the disadvantage of not being able to screen short peptides, which indicates that selecting appropriate targets will help screen more anti-inflammatory peptides. Finally, following the docking results of peptides, anti-inflammatory peptides were further screened and combined with the synthesis cost of peptides. The peptides with the lowest docking energy were synthesized under the same chain length. Table 2 shows the 17 peptides obtained from broccoli, which are mostly derived from water-soluble proteins such as F-box domain-containing protein, rubisco (Supplementary Table S4). The results of the remaining molecularly docked peptides are in Supplementary material C. To further research the anti-inflammatory action of broccoli-derived peptides, these 17 peptides were verified by cell experiments.

**Table 2:** The energy of molecular docking of anti-inflammatory peptides with iNOS.

**Samples**	**Peptide**	**Abbreviation**	**Length**	**-CDOCKER energy (kcal/mol)**	**-CDOCKER interaction energy (kcal/mol)**
NC & *L. plantarum* A3	GDRW	GW-4	4	92.9559	78.0537
	DGRYW	DW-5	5	110.217	72.6409
	AAMVWPPLGK	AK-10	10	133.552	123.125
*L. plantarum* A3	QGAGYRW	QW-7	7	121.395	93.4092
	KASFAFAGL	KL-9	9	116.507	97.8707
	FNFH	FH-4	4	95.6975	73.2568
	MHHYW	MW-5	5	106.761	88.6178
	SIWYGPDRP	SP-9	9	81.799	103.996
	HFKQPW	HF-6	6	114.205	103.551
	FGDFNPGGRL	FL-10	10	129.955	99.2752
*L. rhamnousus* ATCC7469	DPWHNF	DF-6	6	110.896	89.0591
	WKRW	WW-4	4	90.0464	82.2929
NC, *L. plantarum* A3 & *L.rhamnousus* ATCC7469	KWR	KR-3	3	75.5454	66.923
*L. plantarum* A3 & *L. rhamnousus* ATCC7469	RFR	RR-3	3	71.6026	66.1001
	ADLAHLPF	AF-8	8	124.091	96.4943

The abbreviation is composed of the first amino acid + terminal amino acid + peptide chain length.

### 3.5. Effect of synthetic broccoli peptides on the production of NO, TNF-α, and IL-6

**Figure 5:**
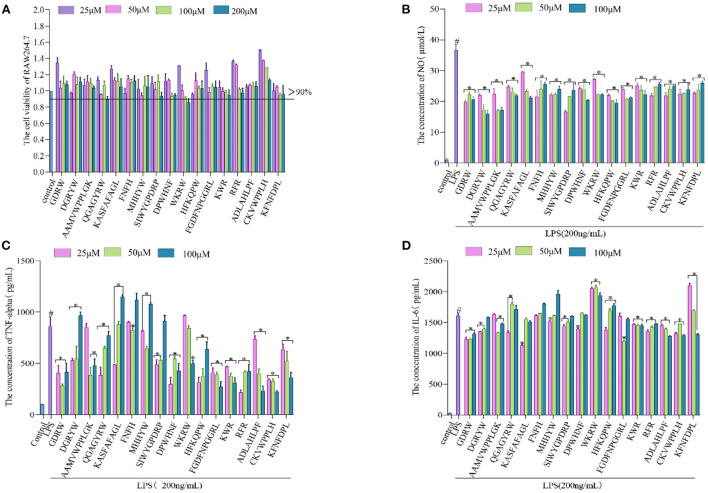
Validation of anti-inflammatory activity of the broccoli-derived synthetic peptide. **(A)** Effect of synthetic peptide on RAW264.7 cell viability. **(B)** Effect of synthetic peptide on NO release from inflammatory cells. **(C)** Effect of synthetic peptide on TNF-α release from inflammatory cells. **(D)** Effect of synthetic peptide on IL-6 release from inflammatory cells. #, LPS is significantly different from control; *, The difference between the experimental group and the LPS group was significant (*P* < 0.05).

The anti-inflammatory action of synthetic broccoli peptides was further verified by using an inflammatory cell model. Based on the cell viability test (Figure 5A), low concentrations (25, 50, and 100 μM) were selected as the test concentrations (cell viability >90%). It is well known that the release of NO is related to the expression of iNOS. When iNOS is inhibited, NO production is reduced, thereby reducing inflammation (4) Therefore, the Griess test was adopted to verify the inhibition of peptides screened by molecular docking on iNOS. The results showed that 17 peptides could inhibit the secretion of NO by macrophages after LPS stimulation. Among them, SIWYGPDRP had the strongest ability to inhibit the release of NO from inflammatory cells at a concentration of 25 μM, with an inhibition rate of 52.32 ± 1.48%. DGRYW reduced NO content in inflammatory cells in a dose-dependent manner. At the concentration of 100 μM, the inhibition rate of NO release was 53.94 ± 2.49% (Figure 5B). This indicates that the peptides may inhibit the production of NO by inhibiting the expression of iNOS to achieve the purpose of treating inflammation. This is identical to the anti-inflammatory effect of hazelnut peptides reported by Ren et al. (43).

When the inflammation generates, iNOS inhibitors can attenuate the inflammatory reaction by restraining iNOS activity. In addition, it can also regulate the inflammatory pathway affecting the release of cytokines to inhibit the inflammatory reaction (50). TNF-α and IL-6 are representative inflammatory cytokines. The ability of 17 peptides to inhibit the secretion of inflammatory factors TNF-α and IL-6 was further explored and shown in Figures 5C, D. It is found that the ability of cells to secrete inflammatory cytokines (TNF-α and IL-6) was significantly enhanced after LPS induction. The production rates of TNF-α and IL-6 were significantly decreased after synthetic peptide treatment. GDRW and ADLAHLPF significantly inhibited the production of TNF-α and IL-6 and relieved the inflammatory response. ADLAHLPF, FGDFNPGGRL, and CKVWPPLH dose-dependently decreased TNF-α. At a concentration of 100 μM, their inhibition rates on TNF-α were 73.19 ± 5.31%, 68.44 ± 5.63%, and 73.83 ± 0.81%, respectively.

In addition, we found that the ability of some peptides to inhibit the release of TNF-α or IL-6 gradually decreased with the increase in concentration, and even there was almost no inhibition rate at high doses. Some peptides showed a U-shaped dose relationship only at medium concentrations. The peptide KASFAFAGL had the strongest inhibitory effect on TNF-α and IL-6 at a low dose, with inhibition rates of 43.59 ± 0.27% and 29.84 ± 0.63%, respectively. However, when its concentration exceeded 25 μM, it did not show inhibitory activity on TNF-α and IL-6 secretion. FGDFNPGGRL only inhibited IL-6 at a concentration of 50 μM. RFR showed the strongest ability to inhibit TNF-α at a concentration of 25 μM, with an inhibition rate of 74.61 ± 1.68%, but this ability gradually weakened as the concentration increased. These phenomena were possibly concerned with cell toleration. Peptides showed an inhibitory effect at a certain dose, but beyond this range, the inhibitory effect weakened or even disappeared. Complex mechanisms of conceivability changes, receptor internalization, and occupancy saturation result in different doses of peptide binding to the target, thereby affecting anti-inflammatory activity. Studies have shown that the peptide KIWHHFF identified from sturgeon protein inhibited LPS-induced IL-6 secretion only at the lowest concentration but did not exhibit TNF-α inhibitory activity (44). Zhao et al. (51) also proved that the peptides identified from antler proteins showed anti-inflammatory activity in a U-shaped dose effect.

Amino acid sequence and position are closely related to peptide function. Previous studies have shown that anti-inflammatory peptides were rich in hydrophobic amino acid residues and positively charged amino acid residues, especially N-terminal or C-terminal (40). Highly hydrophobic peptides can promote the interaction between peptides and cell membranes, thereby regulating downstream pathways and exhibiting anti-inflammatory effects. In addition, one or multiple glutamines, glutamic acid, tyrosine, tryptophan, cysteine, and asparagine acid residues may contribute to the immunoregulatory activity of food protein-derived peptides (52). In addition, peptides containing positively charged amino acid residues (lysine, arginine, and histidine) exert anti-inflammatory effects by binding to LPS released by bacteria. In this study, anti-inflammatory peptides were found to contain one or more hydrophobic amino acids. Such as GDRW, KASFAFAGL, FGDFNPGGRL, and ADLAHLPF were rich in hydrophobic amino acids and aspartic acid, and tryptophan. HFKQPW, RFR, and KWR all had positively charged amino acid residues. To sum up, the constitution and specific location of amino acids might contribute to the anti-inflammatory activity of 17 novel peptides.

### 3.6. Molecular mechanism based on iNOS of anti-inflammatory peptides

**Figure 6:**
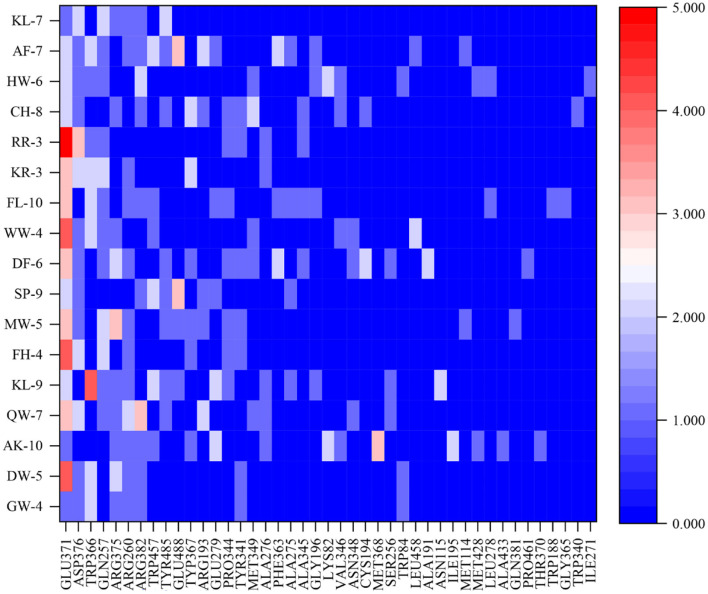
The docking sites and interaction forces of 17 anti-inflammatory peptides with iNOS (The abscissa represents the docking site between the peptide and the receptor, and the ordinate value represents the number of forces between the peptide and the receptor).

Comprehension of the important active sites of anti-inflammatory peptides was of great significance for understanding the recognition mode of ligands and receptors. Figure 6 reveals the interaction between anti-inflammatory active peptides and iNOS receptor binding sites. There were 43 active sites for non-bonded interaction between 17 anti-inflammatory peptides and iNOS receptors. Among them, Glu371, Asp376, Trp366, Gln257, Arg375, Arg260, and Arg382 are the most active sites for non-bond binding with anti-inflammatory peptides.

**Figure 7:**
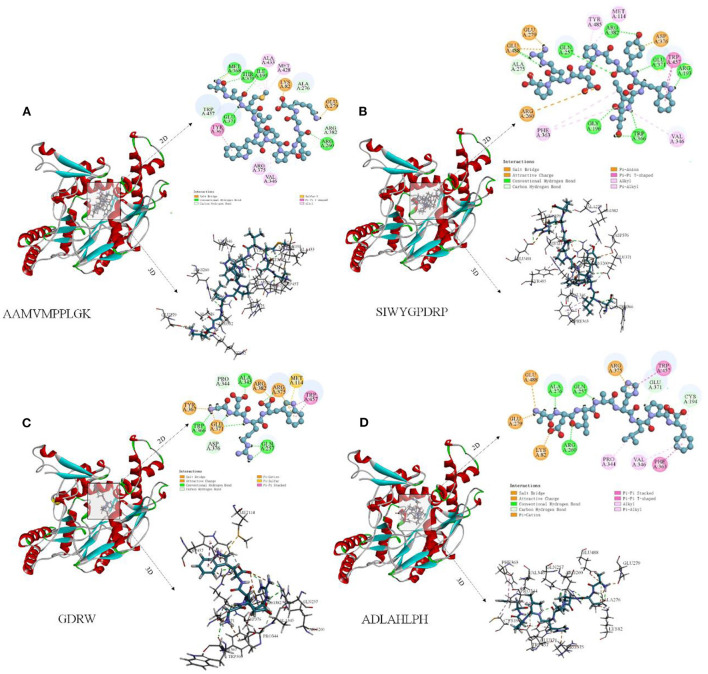
Molecular docking diagram of anti-inflammatory peptide binding to iNOS receptor. **(A–D)** Three-dimensional and two-dimensional spectra of the interaction of some anti-inflammatory peptides with iNOS receptors.

The peptide-iNOS interaction in the spatial pattern of binding force has a great impact on anti-inflammatory activity, including hydrogen bonds (salt bridge, conventional hydrogen bond, carbon-hydrogen bond, and pi-donor hydrogen bond) electrostatic interactions (attractive charge, pi-anion, and pi-cation), hydrophobic interactions (pi-pi T-shaped, pi-sigma, alkyl, and pi-alkyl), and other interactions (37). Figures 7A–D depicts 3D and 2D images of the binding of the anti-inflammatory peptide to the iNOS receptor, showing the most appropriate docking posture. The binding sites of the remaining anti-inflammatory peptides are shown in Supplementary Figure S4. The active sites and forces of 17 anti-inflammatory peptides were analyzed, and it was found that these peptides mainly bind to the iNOS receptor residues through hydrogen bonds and hydrophobic interaction, which was identical to the previous research by Peng et al. (4). The residues in iNOS for the formation of the salt bridge were concentrated on Glu371, Asp376, Arg382, Arg375, and Glu279, while the residues Gln257, Glu371, Trp366, Asp376, Tyr341, Tyr367, Arg260 contributed to the formation of traditional hydrogen bonds. The residues Glu371, Gln257, and Pro344 contributed to the formation of carbon-hydrogen bonds (Supplementary Figure S4 and Figure 7). As described by Cinelli, Do, Miley, and Silverman (53), iNOS has two main domains, C-terminal reductase containing a flavin mononucleotide (FMN) binding subdomain and an N-terminal oxygenase. In the H4B binding pocket of the oxygenase domain, residues Arg375, Trp455, Trp457, and Phe470 are essential for H4B cofactor binding, subsequent dimerization, and enzyme function. When residues Phe831 and Leu832 were metabolized by polar residues (serine and proline), iNOS activity decreased significantly. Anti-inflammatory peptides may bind to amino acid residues (Arg357, Trp457, and Cys194) in the iNOS activity pocket and interact with iNOS and limit iNOS activity (Figure 6).

Peptide AAMVWPPLGK (AK-10) has the lowest docking energy (−133.552 kcal/mol) with iNOS (Table 1). It could be found that Met368, Thr370, Ile195, Glu371, and Arg260 from hydrogen bonds with −C=O, Lys82, Lys82, and Glu279 form hydrogen bonds with −C=O and –NH^3+^ group, respectively, Arg376, Val346, and Met348 formed pi-alkyl bonds (Figure 7A). The peptide SIWYGPDRP with the strongest inhibitory activity on NO release at low concentration has lower docking energy (−88 kcal/mol) with the iNOS receptor (Table 1). As shown in Figure 7B, the interaction between ligand and receptor was mainly hydrogen bond and Alkyl. The N-terminal of SIWYGPDRP interacted with amino acid residues Trp457 and Arg193 through hydrogen bonds and Pi-Pi T-Shaped. In addition, hydrogen bonds were formed with Gly196, Gln257, Trp366, Arg382, and Glu371. In addition to hydrogen bonds, SP-9 also binds to the iNOS receptor cavity through salt bridges, electrostatic interactions, Alkyl, and carbon-hydrogen bonds, thereby inhibiting iNOS enzyme activity. Other anti-inflammatory peptides such as ADLAHLPF and GDRW also demonstrated that they can bind to iNOS receptor, as shown in Figures 7C, D. Hydrogen bond interactions are mainly formed between the oxygen atoms of these anti-inflammatory peptides and the hydrogen atoms of iNOS amino acid residues. Or the hydrogen atom between the amino acid residue and the amino group of the anti-inflammatory peptide forms a hydrogen bond interaction.

## 4. Conclusion

By peptidomic and virtual screening technology, 17 novel anti-inflammatory peptides were identified from *L. plantarum* A3 and *L. rhamnosus* ATCC7469. These anti-inflammatory peptides were mainly derived from water-soluble proteins dominated by F-box domain-containing proteins. These peptides exert anti-inflammatory activity by inhibiting the secretion of inflammatory factors by inflammatory cells. Combined with the molecular docking results, 17 peptides could produce hydrogen bonds and hydrophobic interactions with multiple receptor residues in the iNOS binding pocket. Additionally, molecular docking results showed that Glu371, Asp376, Trp366, Gln257, and Arg375 may play an important role in anti-inflammatory activity by molecular docking with iNOS. This study could provide a reference for the research and utilization of food anti-inflammatory peptides. It is estimated that only 10% of drugs have enough data to prove the safety and effectiveness of their use in pregnant women. This study may provide new ideas for anti-infection during pregnancy in terms of safety and effectiveness.

## Data availability statement

The original contributions presented in the study are included in the article/Supplementary material, further inquiries can be directed to the corresponding authors.

## Author contributions

YL: conceptualization, formal analysis, investigation, methodology, writing–original draft, and visualization. XG: investigation, software, and supervision. DP: project administration and supervision. ZL: visualization, software, and supervision. CX: data curation, funding acquisition, and project administration. YX: methodology, software, data curation, and investigation. LD: supervision. ZC and WL: methodology, investigation, and validation. YD: methodology, supervision, project administration, funding acquisition, and writing–review and editing. XZ: visualization. All authors contributed to the article and approved the submitted version.
