# Supplementary material for: Identification and virtual screening of novel anti-inflammatory peptides from broccoli fermented by Lactobacillus strains
Source: Front Nutr. 2023 Jan 11;9:1118900. doi: 10.3389/fnut.2022.1118900 (PMC9875028; doi:10.3389/fnut.2022.1118900)
Supplement: Supplementary file 1 [file Data_Sheet_1.DOCX]

Supplementary Material

**Identification and Virtual Screening of Novel Anti-inflammatory Peptides from Broccoli Fermented by *Lactobacillus Strains***

**Yao Li^1^, Xinchang Gao^2^, Daodong Pan^1^, Zhu Liu^3^, Chaogeng Xiao^4^, Yongzhao Xiong^1^, Lihui Du^1^, Zhendong Cai^1^, Wenjing Lu^4^, Yali Dang^1,^*and Xiuzhi Zhu^5,*^**

**^*^ Correspondence:**

Yali Dang

dangyali1978@126.com

Xiuzhi Zhu

zhuxiuzhi13142@163.com

# Supplementary Tables and Figures:

Table S1. Peptide desalination steps

| Step | concrete operation |
| --- | --- |
| 1 | 100 % acetonitrile elution |
| 2 | Equilibrium of 0.1% Trifluoroacetic Acid Aqueous Solution |
| 3 | Add peptide sample |
| 4 | Elution of salt and other impurities with 0.1 % trifluoroacetic acid aqueous solution |
| 5 | 50 % acetonitrile 0.1 % trifluoroacetic acid aqueous solution eluted the polypeptide from the column |
| 6 | freeze drying |

Table S2. Methods for determination of inflammatory factors

| Assay | Method |
| --- | --- |
| Determination of NO concentration | 50μL cell culture supernatant was mixed with 50μL griess I and 50μL griess II at room temperature for 5 min. 540 nm out read absorbance. Different Concentrations of NaNO_2_ as Standard for Calculating NO Concentration. |
| Determination of TNF-α and IL-6 concentration | Test according to manufacturer 's instructions. Finally, dark color 10min, 450nm out, 630nm reference wavelength to read the absorbance. Calculate the concentration according to the standard curve. |

Table S3 Energy of molecular docking between known anti-inflammatory peptides and iNOS

| Peptides | Lenth | -CDOCKER ENERGY(kcal/mol) | -CDOCKER INTERACTION ENERGY(kcal/mol) | Peptides | Lenth | -CDOCKER ENERGY | -CDOCKER INTERACTION ENERGY |
| --- | --- | --- | --- | --- | --- | --- | --- |
| APD | 3 | 54.9125 | 44.4115 | LLEL | 4 | 92.0743 | 64.6054 |
| APTLW | 5 | 74.8957 | 56.6525 | LLLE | 4 | 91.6038 | 65.199 |
| AVGPAGPRG | 9 | 100.269 | 88.9421 | LPF | 3 | 47.6339 | 43.5508 |
| DEDTQAMPF | 9 | 141.421 | 103.115 | LREMLSTMCTARGA | 14 | 182.124 | 130.359 |
| DEDTQAMPFR | 10 | 147.208 | 128.208 | LSPLLAAH | 8 | 119.202 | 100.867 |
| DNIQGITKPAIR | 12 | 149.784 | 135.679 | LTGP | 4 | 62.3087 | 52.209 |
| DTQAMPFR | 8 | 117.918 | 85.3835 | LVGKLLKGAVGDVCGLLPIC | 20 | 148.531 | 122.858 |
| EDDQMDPMAK | 10 | 160.105 | 131.928 | LVYPFPGPI | 9 | 88.6988 | 107.559 |
| EGLLGDVF | 8 | 145.2 | 98.0026 | LY | 2 | 56.2929 | 48.1417 |
| FDKPVSPLL | 9 | 118.156 | 114.036 | MGPAMMRTMPG | 11 | 105.237 | 110.529 |
| FLWGKSY | 7 | 120.58 | 101.79 | MLGATSL | 7 | 110.364 | 81.0092 |
| FSGLDGAKGD | 10 | 158.28 | 107.444 | MMLDF | 5 | 99.5624 | 82.4644 |
| GHS | 3 | 65.4121 | 44.2799 | MSYSAGF | 7 | 114.771 | 88.7616 |
| GPAGPL | 6 | 60.2602 | 56.5278 | NCWPFQGVPLGFQAPP | 16 | 111.98 | 150.228 |
| GPETAFLR | 8 | 118.986 | 97.7067 | NPAQDC | 6 | 96.3215 | 65.4966 |
| GPPGAP | 6 | 50.1295 | 52.3817 | PAY | 3 | 47.7597 | 59.2957 |
| GVYY | 4 | 81.7171 | 62.3189 | PRRTRMMNGGR | 11 | 113.668 | 131.15 |
| HK | 2 | 68.3853 | 61.8077 | QA | 2 | 55.9625 | 42.832 |
| HKGQCC | 6 | 109.945 | 74.1305 | QCQQAVQSAV | 10 | 151.829 | 104.265 |
| HLDDALRGQE | 10 | 167.726 | 122.692 | QNWDFCEAWEPCF | 13 | 175.288 | 150.766 |
| IAFKTNPNSMVSHIAGK | 17 | 157.961 | 142.788 | RALP | 4 | 69.3351 | 55.7931 |
| IDVSPDSPDHY | 11 | 148.638 | 138.361 | SDIKHFPF | 8 | 135.237 | 104.789 |
| IGVAMDYSASSKR | 13 | 154.243 | 138.098 | SDLKHFPF | 8 | 140.583 | 110.591 |
| IPAV | 4 | 56.6138 | 54.9147 | SLSFASR | 7 | 105.476 | 97.9302 |
| IQDKEGIPPDQQR | 13 | 108.31 | 118.575 | SLVNNDDRDS | 10 | 182.505 | 135.441 |
| IRW | 3 | 69.0819 | 69.1685 | TVNLAYY | 7 | 132.09 | 97.0459 |
| IVPAS | 5 | 71.1863 | 53.0747 | VAPAWGPWPKG | 11 | 93.2097 | 104.224 |
| IYVDAVINH | 9 | 155.77 | 115.787 | VDVPVKVPYS | 10 | 121.124 | 117.72 |
| KA | 2 | 54.2624 | 37.0009 | VGPAGPAGP | 9 | 68.1429 | 80.6493 |
| KIWHHTF | 7 | 134.302 | 89.9842 | VHYAG | 5 | 90.1686 | 77.1702 |
| KLPDHPKLPK | 10 | 123.588 | 129.998 | VIESPPEI | 8 | 96.6119 | 94.4476 |
| KQSESHFVDAQPEQQQR | 17 | 195.835 | 173.056 | VVHF | 4 | 85.1237 | 63.0463 |
| KVLPVPEK | 8 | 116.243 | 94.0219 | WFNNAGP | 7 | 91.7285 | 80.9597 |
| LDAPGHR | 7 | 111.972 | 98.5747 | WG | 2 | 49.3348 | 44.576 |
| LDAVNR | 6 | 124.727 | 86.2233 | YPW | 3 | 52.5605 | 55.6137 |

Table S4 Source of identified anti-inflammatory peptides.

| Samples | Peptide | Abbreviation | Lenth | -CDOCKER Energy (kcal/mol) | -CDOCKER Interaction Energy (kcal/mol) | Sources |
| --- | --- | --- | --- | --- | --- | --- |
| NC+ *L.plantarum* A3 | GDRW | GW-4 | 4 | 92.9559 | 78.0537 | F-box domain-containing protein |
|  | DGRYW | DW-5 | 5 | 110.217 | 72.6409 | Ribulose bisphosphate carboxylase small subunit |
|  | AAMVWPPLGK | AK-10 | 10 | 133.552 | 123.125 | denovo |
| *L.plantarum* A3 | QGAGYRW | QW-7 | 7 | 121.395 | 93.4092 | denovo |
|  | KASFAFAGL | KL-9 | 9 | 116.507 | 97.8707 | Peroxidase |
|  | FNFH | FH-4 | 4 | 95.6975 | 73.2568 | Transmembrane protein 18 |
|  | MHHYW | MW-5 | 5 | 106.761 | 88.6178 | denovo |
|  | SIWYGPDRP | SP-9 | 9 | 81.799 | 103.996 | Chlorophyll a-b binding protein |
|  | HFKQPW | HF-6 | 6 | 114.205 | 103.551 | Malic enzyme |
|  | FGDFNPGGRL | FL-10 | 10 | 129.955 | 99.2752 | Uncharacterized protein |
| *L.rhamnousus* ATCC7469 | DPWHNF | DF-6 | 6 | 110.896 | 89.0591 | denovo |
|  | WKRW | WW-4 | 4 | 90.0464 | 82.2929 | ADP/ATP translocase |
| NC+*L.plantarum* A3+*L.rhamnousus* ATCC7469 | KWR | KR-3 | 3 | 75.5454 | 66.923 | Aldehyde dehydrogenase  50S ribosomal protein L22  F-box domain-containing protein |
| *L.plantarum* A3+*L.rhamnousus* ATCC7469 | RFR | RR-3 | 3 | 71.6026 | 66.1001 | F-box domain-containing protein  40S ribosomal protein S4 |
|  | ADLAHLPF | AF-8 | 8 | 124.091 | 96.4943 | Glutathione transferase |


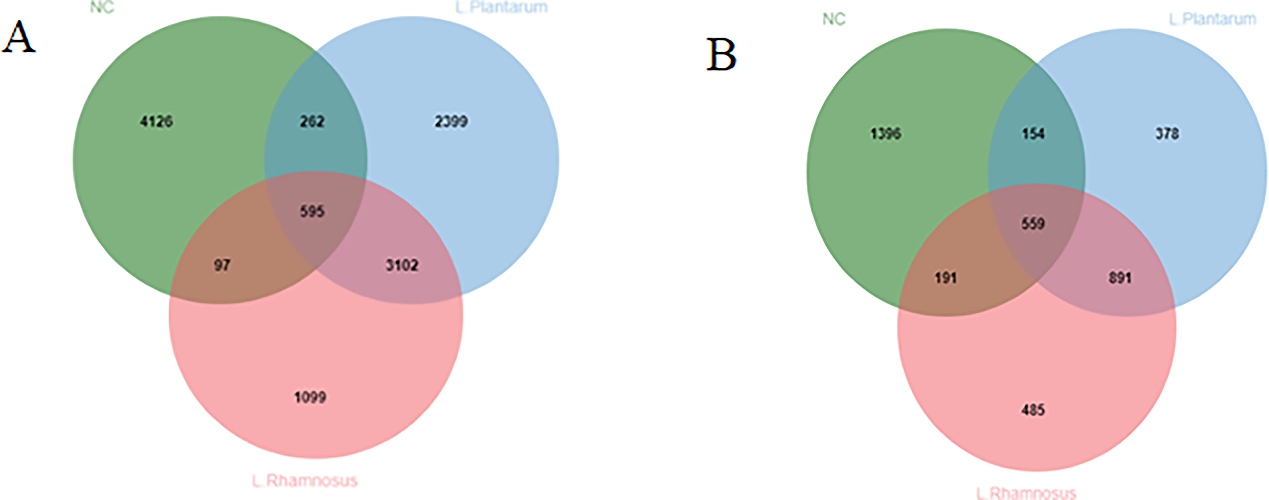


Figure S1. Venn diagrams of identified peptides from database (a) and proteins (b) (lower than 10 kDa) from different broccoli fermentated samples.


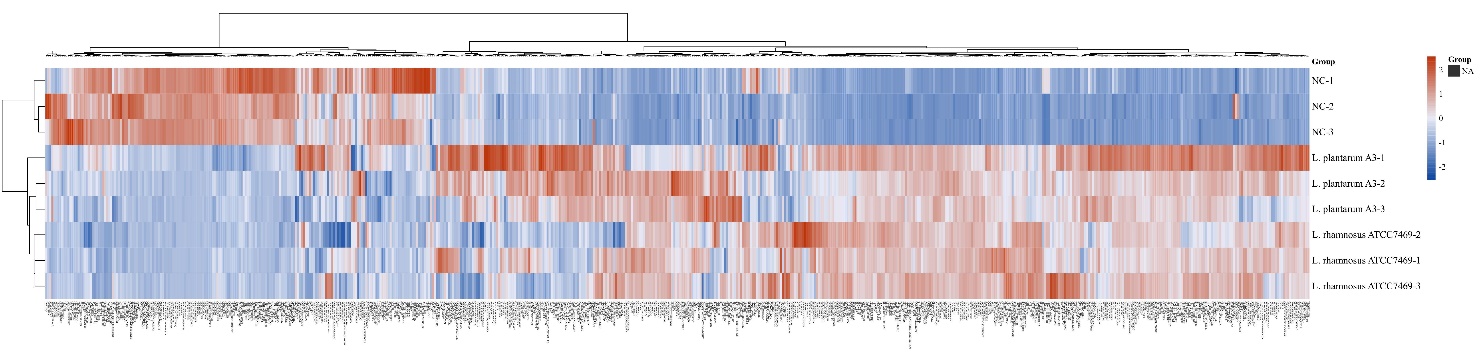
Figure S2. Heat map and hierarchical clustering of common peptides in three samples.


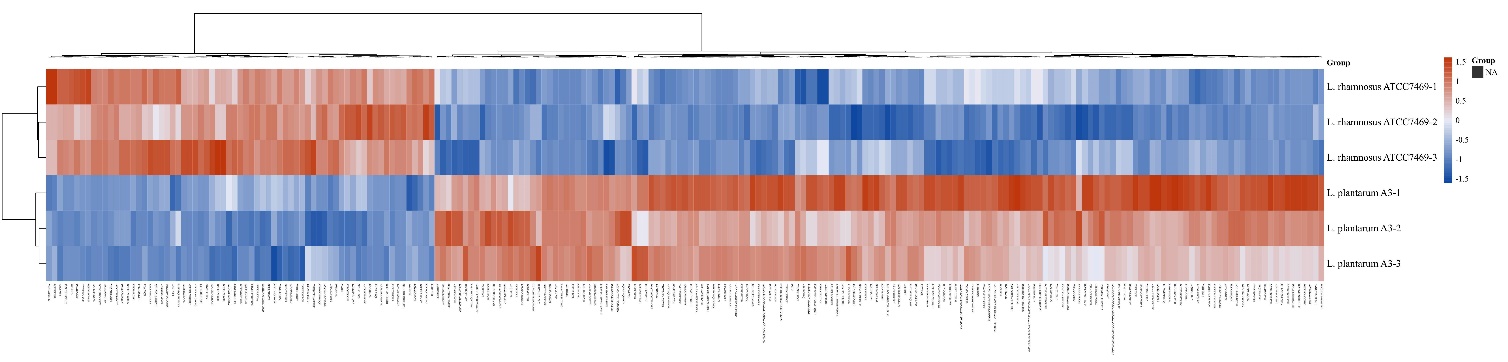
Figure S3. Heatmap and hierarchical cluster analysis of 270 significantly different peptides that co-existed in both samples but not in NC.


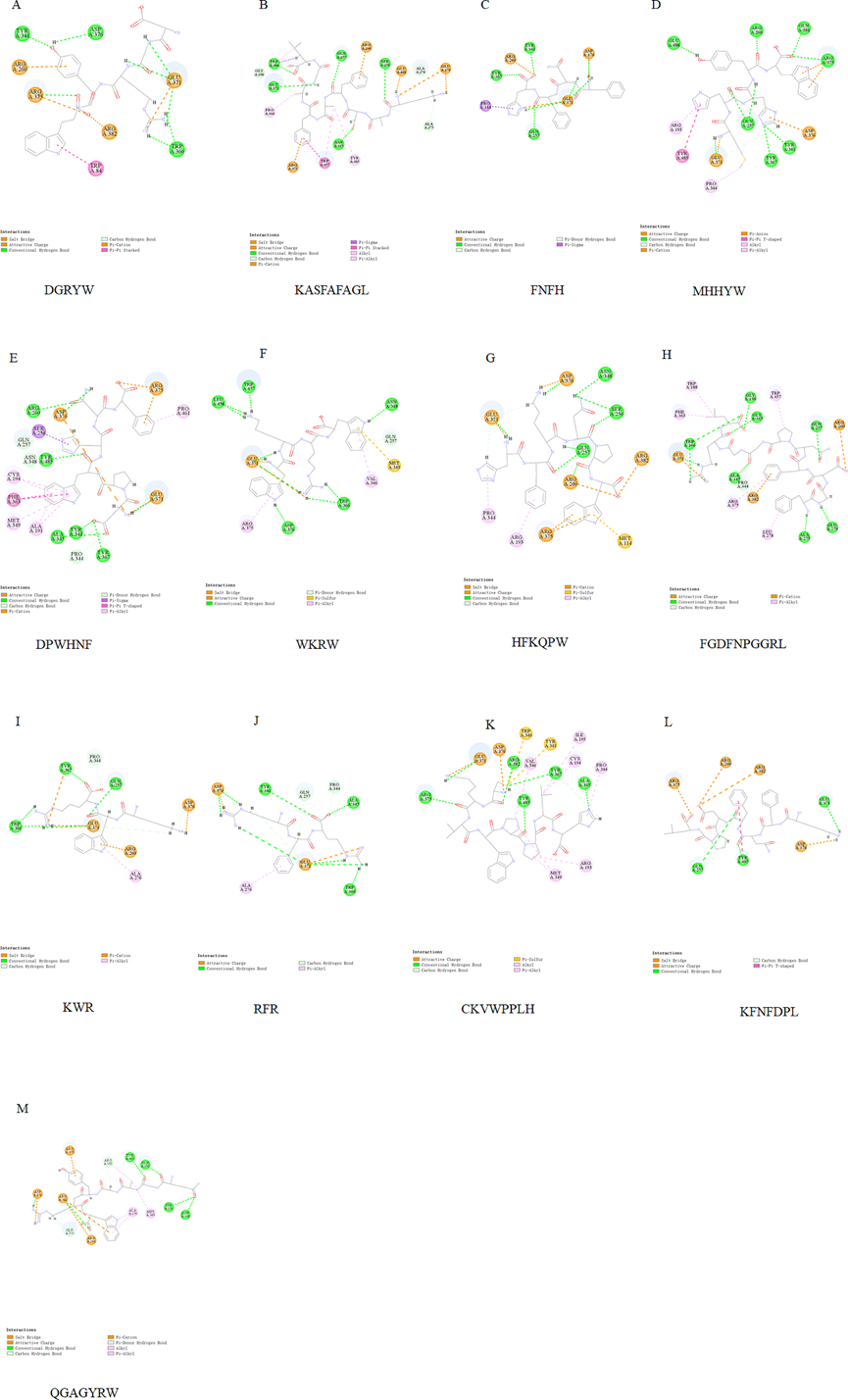


Figure S4. 2D diagram of optimal conformation of anti-inflammatory peptide and iNOS receptor.
